# Research on auditory and olfactory regulation methods for abnormal driver emotions based on EEG signals

**DOI:** 10.3389/fnhum.2025.1615346

**Published:** 2025-06-16

**Authors:** Bangbei Tang, Yan Li, Yingzhang Wu, Yilun Li, Qizong Yue

**Affiliations:** ^1^School of Intelligent Manufacturing Engineering, Chongqing University of Arts and Sciences, Chongqing, China; ^2^Department of Physiology, Army Medical University, Chongqing, China; ^3^School of Vehicle and Mobility, Tsinghua University, Beijing, China; ^4^School of Music and Dance, Henan Institute of Science and Technology, Xinxiang, China; ^5^China Music Mental Health Institute, Southwest University, Chongqing, China

**Keywords:** brain, driving emotions, music intervention, emotional regulation, fragrance intervention

## Abstract

**Introduction:**

In sudden and dangerous traffic situations, drivers are susceptible to abnormal emotional states, such as tension and anger, which can significantly increase safety risks while driving. Electroencephalography (EEG) signals, being an objective measure of emotional states, offer valuable insights for identifying and regulating these emotions.

**Methods:**

This study collected EEG data from 54 drivers in a simulated driving environment, resulting in a total of 1,260 samples, and developed a recognition model for abnormal emotions—specifically tension and anger—based on the EEG signals. Time-frequency domain features, including mean, variance, skewness, kurtosis, root mean square, and power spectral density, were extracted and analyzed using classification algorithms such as Back Propagation Neural Networks (BPNN), K-Nearest Neighbors (KNN), and Support Vector Machines (SVM), enabling precise identification of varying levels of tension and anger. Furthermore, the study assessed the effects of music, fragrance, and their combined application on alleviating these abnormal emotional states.

**Results:**

Results indicated that music, fragrance, and their combination were related to a reduction in stress and anger across different severity levels, with subjective assessments correlating well with the objective EEG data. Notably, music regulation was found to be most effective for mild and moderate tension, reducing tension levels by 63.33% and 68.75%, respectively, whereas fragrance was more efficacious in high tension situations, achieving a 43% reduction. For anger, fragrance regulation proved more beneficial for mild and moderate anger (reducing anger by 66.67 and 73.75%, respectively), while music regulation was most effective in mitigating high anger levels, resulting in a 58% reduction. Additionally, an analysis of time-domain features utilizing Hjorth parameters revealed that the application of a single fragrance was most effective for alleviating tension, while a singular music regulation strategy demonstrated superior performance in calming anger.

**Discussion:**

The reliability of both the abnormal emotion recognition model and the emotion regulation assessment system was validated through the study. These findings contribute valuable scientific evidence for the management of drivers’ emotions and suggest promising avenues for optimizing personalized emotional regulation strategies in the future.

## 1 Introduction

Emotions play a crucial role in influencing driving behavior (Hu et al., 2022). Drivers are particularly susceptible to experiencing abnormal emotional states (Conceição et al., 2023; Lin et al., 2022; Xi et al., 2022), such as stress (Han et al., 2024; Mukherjee and Anwaruzzaman, 2024) and anger (Âbele et al., 2020; Celiñski, 2022), which may arise from various factors including complex road conditions, traffic congestion, and time pressures. These negative emotions not only compromise drivers’ mental health but also pose significant threats to driving safety, thereby greatly increasing the risk of traffic accidents (Habibifar and Salmanzadeh, 2022; Li G. et al., 2023). Consequently, the effective identification and regulation of abnormal emotions in drivers have emerged as critical areas of research aimed at enhancing overall driving safety.

Electroencephalography (EEG) serves as an intuitive reflection of the physiological states of drivers’ brains (Peng et al., 2022), effectively capturing their emotional states (Li J. et al., 2023). Numerous researchers have explored the relationship between EEG signals and emotions. Atkinson et al. (Atkinson and Campos, 2016) extracted time-domain features, including median, standard deviation, and kurtosis, to facilitate emotion recognition using the DEAP dataset. Khalili and Moradi (2009) utilized average, variance, skewness, and peak values of EEG signals to identify positive, negative, and neutral emotional states. Hasan et al. (2021) employed various film clips to elicit five common emotional states—calmness, joy, sadness, tension, and disgust—achieving a maximum recognition rate of 89.22% for classifying EEG signals associated with these emotions. Wagh and Vasanth (2022) categorized EEG signals into alpha, beta, delta, theta, and gamma frequency bands and combined these with machine learning algorithms for feature extraction, enabling emotion classification, recognition, and association analysis. Li et al. (2018) extracted time-frequency domain features, such as arithmetic mean, root mean square, power spectral density, power spectral entropy, singular spectrum entropy, and approximate entropy, from EEG signals in the SEED dataset, achieving a cross-validation classification accuracy of 83.33% with their Support Vector Machine (SVM) emotion recognition model. Subasi et al. (2021) introduced an ensemble learning method that integrates Random Forest (RF) and SVM, with results demonstrating that this ensemble approach outperformed single classifiers across various datasets. Wu et al. (2023) presented an emotion recognition method using only the Fp1 and Fp2 channels of frontal lobe EEG signals, employing a Gradient Boosting Decision Tree (GBDT) classifier to empirically validate the effectiveness of this approach, achieving an average classification accuracy of 75.18%.

Most current research focuses on the correlation between EEG signals and emotions, while investigations into emotional regulation management remain relatively limited. Studies examining the alleviation of abnormal emotions through olfactory or auditory stimuli are particularly scarce. Although existing literature indicates that certain odors and musical stimuli can elicit specific emotional responses (Chaichanasittikarn et al., 2023; Laktionova et al., 2024; Pring et al., 2024; Putkinen et al., 2024), many of these studies predominantly provide superficial descriptions and lack a thorough exploration of the underlying neurobiological mechanisms. Furthermore, there is a significant gap in effective methods for identifying abnormal emotions, as well as a lack of robust assessment models to evaluate the efficacy of emotion regulation strategies, especially in the context of driving environments.

Building on this foundation, the present study developed a model to identify driver stress and anger using EEG signals. Furthermore, it systematically evaluated the effects of various emotional regulation strategies—including music, fragrance, and their combined application—on the modulation of abnormal emotions in drivers. The findings provide a scientific basis and practical guidance for managing driver emotions, with the aim of reducing the incidence of traffic accidents and minimizing associated losses. Additionally, this study establishes a theoretical framework for future personalized interventions targeting driver emotions, thereby advancing the application of emotion recognition technologies in intelligent transportation systems and facilitating data-driven decision-making for enhanced driving safety.

The primary contributions of this work can be summarized as follows:

(1) This study presents a novel perspective on traffic safety management by integrating EEG signals with musical and olfactory interventions, thereby establishing a foundation for data-driven emotional management decisions. Particularly within the framework of intelligent transportation systems, interventions tailored to the emotional states of drivers have the potential to significantly improve traffic safety and mitigate accidents associated with emotional instability.
(2) This study meticulously distinguishes between varying levels of driver stress (slight, moderate, and high) and anger (slight, moderate, and high), and assesses the effects of soothing music and lemon fragrance on these emotional states. This refined approach enhances the precision and personalization of interventions, thereby offering a robust theoretical foundation for the development of future personalized intervention strategies tailored to driver emotional states.
(3) This study assessed the classification performance of three models in identifying driver stress and anger. The results indicate that, compared to the K-Nearest Neighbors (KNN) and Support Vector Machine (SVM) models, the Back Propagation Neural Network (BPNN) model exhibited significantly higher accuracy in classifying both stress and anger emotional states.

## 2 Materials and methods

### 2.1 Screening of emotional stimuli materials

This study utilized a video-guided experimental paradigm that enhances participant immersion and attentiveness during signal collection. By integrating visual and auditory stimuli, this approach intensifies emotional induction and prolongs the duration of emotional experiences, ultimately leading to the generation of higher-quality EEG signals.

The videos were filmed from a first-person perspective to simulate a realistic driving experience. The videos designed to induce driving stress included scenarios such as high-speed driving, extreme weather conditions, and emergency avoidance maneuvers. In contrast, the videos aimed at eliciting anger portrayed situations such as traffic congestion, traffic violations, and provocations from other drivers. To mitigate potential loss of interest among participants due to excessive video duration, which could negatively impact the emotional induction process, the length was intentionally limited to 3 min, thereby helping to sustain participants’ attention and engagement (Xu et al., 2024).

Prior to the experiment, videos intended to induce stress and anger were meticulously screened. The video materials were sourced from the internet. Participants’ emotional states were assessed using a five-point Likert scale immediately after viewing the inducing videos, with a score of 3 indicating successful emotional induction. Specifically, a score of 1 represented “Calm,” a score of 2 indicated “Almost not,” a score of 3 indicated “Slight,” a score of 4 represented “Moderate,” and a score of 5 indicated “High.” Only videos with an induction success rate exceeding 80% were selected as emotional stimuli. The Likert scale used in this study is presented in Table 1.

**TABLE 1:** Likert level 5 scale.

Likert scale					
Emotion	Calm (1)	Almost not (2)	Slight (3)	Moderate (4)	High (5)
Stress	°	°	°	°	°
Anger	°	°	°	°	°

### 2.2 Experimental scenario

Given that this experiment requires participants to drive while experiencing altered emotional states—where real-world driving presents significant safety risks and operational challenges—this study employed a driving simulation experiment as a viable alternative. The primary advantages of this approach include enhanced safety, precise control over experimental conditions (such as temperature, lighting, and audio), and reduced costs. Additionally, driving simulators can effectively replicate real driving scenarios, thereby ensuring the reliability and validity of the experimental results. Numerous studies have shown that the physiological responses observed in driving simulations closely resemble those in actual driving environments (Bobermin et al., 2021). Consequently, this study utilizes driving simulators instead of real-world driving.

This study utilized the Forza Horizon 5 software developed on the EA platform, in conjunction with the Laisida V99 driving simulator, to create a simulated driving environment. The simulator effectively replicates real-world traffic conditions and comprises three main components: the vehicle operation system, the visual display system, and the audio system. The vehicle operation system includes a steering wheel, gear shifter, accelerator pedal, brake pedal, and clutch. The visual display system features an LCD monitor that provides a first-person perspective, while the audio system offers immersive surround sound effects to enhance the simulated driving experience.

**FIGURE 1:**
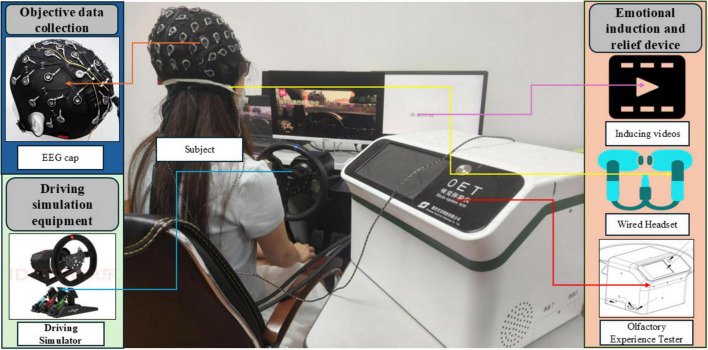
Experimental scenario.

The experimental setting is illustrated in Figure 1. The laboratory features adequate ventilation and sufficient lighting.

The emotional induction videos were presented using E-Prime (Richard and Charbonneau, 2009; Verdonschot et al., 2019), which is integrated with the EEG data collection module to form a synchronized data acquisition system. The electrode placements on the EEG cap followed the international 10-10 system, with CPz and the End electrodes serving as the reference and ground, respectively. During the experimental tasks, electrode impedance was consistently maintained below 5 kΩ. Additionally, the regulating music was played through headphones connected to E-Prime, while olfactory stimulation was delivered using an olfactory experience testing device.

The music used for the regulation is titled “Rose Petals.” This gentle piano piece is set in a major key and features a slow, soft rhythm, with a 30-s excerpt used. Soft music is effective in alleviating negative emotions (Liao, 2024; Xiao et al., 2024). The fragrance used is lemon fragrance, which promotes positive emotions and inner calm while soothing the nervous system (Godfrey, 2018; Smith and Nicholson-Lord, 2024). The current experiment also innovatively combines the soothing melodies of soft music with the refreshing lemon fragrance to investigate their synergistic effects on regulating emotional states.

### 2.3 Participant

This study employed a rigorous three-phase screening process to recruit 54 drivers, consisting of 27 males and 27 females, aged between 20 and 27 years. Participants with pre-existing central nervous system disorders, rhinitis, or auditory impairments were excluded through subjective assessment tests to minimize potential confounding factors. Additionally, individuals who had participated in similar experimental settings previously were also excluded to ensure the selection of appropriate candidates. Before the study commenced, all participants signed an informed consent form that outlined the study’s objectives and the specific tasks they would be required to undertake. The 54 drivers were divided into two groups: the first group of 27 (Female:15, Male:12.) individuals underwent a stress emotion regulation experiment, while the second group of 27 (Female:12, Male:15.) individuals participated in an anger emotion regulation experiment. Comprehensive information regarding the participants is presented in Table 2.

**TABLE 2:** Information of participants.

Female	Male	Age	Driving experience
27	27	20–27 (Mean = 23.24, std = 2.32)	1–9 (Mean = 4.74, std = 2.67)

### 2.4 Experimental procedure

The stress emotion regulation experiment was conducted first. Prior to the formal initiation of the experiment, participants were given 30 s to calm their mood, during which their EEG signals were recorded in this relaxed state. Following the calming phase, participants viewed a stress-inducing video. After 60 s of video playback, the experimenters assessed participants’ perceived stress levels using a five-point Likert scale. If participants reported a stress level of 3 (slight stress) or higher, their EEG signals were recorded for the subsequent 30 s. After this, a regulation phase lasting 30 s was conducted, during which additional EEG data were collected. Each stage—calm, induction, and regulation—was clearly defined. A total of 27 participants took part in the stress emotion regulation experiment, divided into three groups of nine. Each group experienced a different regulatory modality: fragrance, music, or a combination of both. Throughout the regulation phase, the stress-inducing video continued to play, and participants’ stress levels were reassessed using the Likert scale after the regulation phase concluded. The results of this assessment were subsequently documented.

Following the completion of the stress emotion regulation experiment, the anger emotion regulation experiment was conducted in a similar manner. The grouping information is presented in Table 3.

**TABLE 3:** Grouping information table.

Emotion	Regulation type	Number of people
Stress	Music	9 (Female:6, Male:3.)
	Fragrance	9 (Female:5, Male:4.)
	Music and Fragrance	9 (Female:4, Male:5.)
Anger	Music	9 (Female:5, Male:6.)
	Fragrance	9 (Female:3, Male:4.)
	Music and Fragrance	9 (Female:4, Male:5.)

**FIGURE 2:**
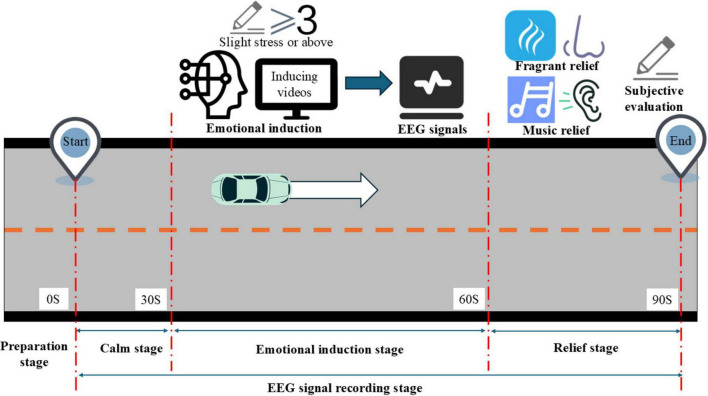
Experimental process.

The experimental process is shown in Figure 2. This figure illustrates the sequence of events in the experiment, including the preparation stage, the calm stage, the emotional induction stage, the emotion regulation experiments carried out by the participants, and the subsequent emotional state assessments. Each stage is clearly depicted, allowing for a better understanding of how the study was conducted and the specific procedures followed by the participants. The right-pointing arrow signifies that the experiment proceeds from left to right.

### 2.5 Ethical approval and compliance statement

This study was approved by the Ethics Committee of Chongqing University of Arts and Sciences (Approval No. CQWL202424). All procedures involving human participants were conducted in accordance with the ethical standards of the 2024 Helsinki Declaration and its subsequent amendments, as well as applicable national and regional regulations. Prior to implementation, the research protocol underwent rigorous review by an independent institutional review board. Written informed consent was obtained from all participants, explicitly addressing the following aspects: (1) participation was voluntary, with the right to withdraw at any stage without penalty; (2) personal information was kept strictly confidential through data anonymization techniques; (3) research data were securely stored in encrypted formats accessible only to authorized investigators; and (4) collected data were exclusively used for the stated scientific purposes.

### 2.6 Data processing and analysis

The machine learning workflow comprises three main stages: data preprocessing, feature extraction, and classification (Alazrai et al., 2019). Feature extraction focuses on capturing both time-domain and frequency-domain characteristics of the signals. Ultimately, the selected features are input into classification algorithms to derive the classification results.

#### 2.6.1 Data preprocessing

**FIGURE 3:**
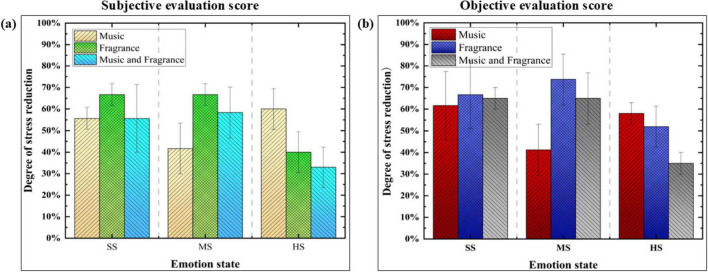
Scores of anger emotion regulation based on subjective and objective data. SA represents “Slight Anger,” MA represents “Moderate Anger,” and HA represents “High Anger.” **(a)** Indicates the subjective evaluation score. **(b)** Indicates the objective evaluation score.

The data exported from the EEG signal acquisition and analysis software comprises signals recorded directly from the scalp, which often contain various types of noise and artifacts. Consequently, preprocessing and denoising are critical steps. EEG artifacts can be classified into two categories: physiological artifacts and non-physiological artifacts. Physiological artifacts typically arise from blinks, eye movements, breathing, and muscle contractions (Islam et al., 2016). In contrast, non-physiological artifacts primarily result from environmental interference, with electrical interference being the most common. The main preprocessing methods for removing artifacts from EEG signals include filtering, referencing, segmenting, and ICA-based artifact removal (Pedroni et al., 2019). The workflow for preprocessing the EEG signals is illustrated in Figure 3. Because the frequency bands associated with driving fatigue overlap with alpha, theta, beta, and delta waves, low-pass filtering at 30 Hz and high-pass filtering at 1 Hz were applied (Sikander and Anwar, 2018). A global average reference was selected, and the data were down sampled to 125 Hz. The preprocessed physiological data were then segmented, specifically extracting data from 0 to 5 seconds post-mark at 0.5-second intervals.

#### 2.6.2 Feature extraction of EEG signals

After preprocessing, the quality of the EEG signal data significantly improved. However, due to the complex nature of EEG signals, which consist of a mixture of various waveforms, it is often necessary to transform the data into a format suitable for statistical analysis during experimental evaluation (Das et al., 2023). Feature extraction is then performed on the preprocessed EEG signals to identify characteristics related to abnormal driving emotions. Given the close relationship between the prefrontal cortex and emotional cognition (Dixon et al., 2017), only data from the prefrontal electrodes FP1, FP2, and FPZ were selected for analysis.

(1) Time-domain Features: Time-domain features of EEG signals play a crucial role in the feature extraction module. EEG signals are time-series signals, and the EEG time-series waveform contains a wealth of time-domain information. Extracting time-domain features of EEG signals is common in brain fatigue detection. Due to their simple calculation and ease of understanding, they are often used to analyze the state of the brain.

(1) Mean Value: The mean value of all sampled values in the EEG signal, reflecting the overall level of the signal

(2) Variance: The average of the squares of the differences between all sampled values of the EEG signal and their mean, reflecting the degree of fluctuation in the signal.

(3) Skewness: This feature is used to measure the asymmetry of the statistical data distribution. Skewness is defined using the third central moment and the second central moment (variance), with the calculation formula as follows:

(4) Kurtosis: It is used to describe the steepness or flatness of the distribution of all values, and its definition is as follows:

(5) Root Mean Square (RMS) is calculated as the square root of the average of the squared values of all EEG signal samples. It is commonly used to quantify the amplitude of EEG signals and provides an indication of the overall amplitude of the signal.

(2) Frequency-domain Features: These refer to the distribution of energy of the EEG signal at different frequencies. Changes in the signal can be obtained from changes in frequency bands, which is the main advantage of frequency-domain analysis compared to time-domain analysis.

(3) Power Spectral Density (PSD): It represents the signal power per unit frequency band and is used to describe the distribution pattern of a signal as it varies with frequency within a certain region. It is a way to study signals from an energy perspective. Generally, the Fourier transform is used to convert EEG signals into frequency-domain signals within a specified frequency band. Power Spectral Density is the most common frequency-domain feature of signals.

Time-domain features capture the dynamic changes and fluctuations of EEG signals, while frequency-domain features reveal the energy distribution of brain signals across different frequencies. During states of stress, both the temporal variations and frequency components of brain signals are impacted. Utilizing both time-domain and frequency-domain features enables a more comprehensive representation of emotional changes, thereby enhancing the accuracy of classification models.

#### 2.6.3 Establishment of a classification model based on EEG signals

After selecting a feature subset with the highest information content, the next step is to learn the mapping function between the features and class labels. This study employed three classification algorithms: K-Nearest Neighbors (KNN) (Shashidhar et al., 2023), Support Vector Machine (SVM) (Son and Kim, 2021), and Backpropagation Neural Network (BPNN) (Sari et al., 2019).

KNN is a straightforward supervised learning algorithm that identifies the K nearest neighbors by calculating the distance between the sample to be classified and the training samples. It classifies the sample based on majority voting among these neighbors, making KNN easy to implement and well-suited for small datasets.

SVM is a robust algorithm designed for binary classification, which seeks the optimal hyperplane to maximize the margin between different classes. By utilizing kernel techniques, SVM effectively handles non-linear problems and can identify linearly separable solutions in high-dimensional spaces.

As a multilayer feedforward neural network, the BPNN possesses robust non-linear modeling capabilities (Zhang et al., 2019), making it well-suited to capture the complex relationships inherent in emotional states, which are influenced by various factors and characterized by high non-linearity. Furthermore, the model continuously adjusts its weights through the backpropagation algorithm, allowing for gradual optimization (Bai et al., 2021). This process enables the model to adapt to different input data and enhances its recognition ability as the number of training samples increases. Through this continuous learning capacity, the BPNN effectively extracts valuable emotional information from extensive EEG signal datasets.

The dataset used for this research consisted of two groups, each containing 630 data samples. The first group encompassed five categories: calm, nearly stress-free, slight stress, moderate stress, and high stress, with 270 samples in the calm category and 90 samples for each of the other levels. The second group also included 630 samples for the categories: calm, nearly anger-free, slight anger, moderate anger, and high anger, with 90 samples for each of the four anger levels. The classification models for abnormal emotions were developed using MATLAB.

Organize the information as shown in Table 4.

**TABLE 4:** Overview of emotion recognition classification model based on EEG features.

Emotion	label	Number of samples	Extracted features	EEG characteristic channel	The algorithm used
Stress	Calm	270	**Time Domain:** Mean, variance, skewness, kurtosis, root mean square, Root Mean Square **Frequency Domain:** Power Spectral Density	**Frontal pole:** FP1, FP2, FPZ	KNN, SVM, BPNN
	Almost no-stress	90			
	Slight stress	90			
	Moderate stress	90			
	High stress	90			
Anger	Calm	270			
	Almost no-anger	90			
	Slight anger	90			
	Moderate anger	90			
	High anger	90			

#### 2.6.4 Model evaluation

This study evaluates the performance of the classification models for recognizing drivers’ abnormal emotional states, designating the model with the highest overall score as the final model. Four assessment metrics were considered: accuracy, precision, recall, and F1 score.

#### 2.6.5 Hjorth parameter analysis and validation

In this study, we conducted a Hjorth parameter analysis on the EEG signals from each stage to assess the effects of music and fragrance on emotional regulation. The Hjorth parameters, which include Activity, Mobility, and Complexity, reflect the energy of the signal, the dispersion of frequency distribution, and the complexity of the waveform, respectively.

For the purpose of analysis, we combined different levels of stress (slight, moderate, high) into a single category labeled “stress,” as well as combining slight, moderate, and high anger into a category labeled “anger.” Through time-frequency analysis of the combined signals, we further validated the effectiveness of music and fragrance in regulating emotional states. This analytical approach helps reveal the impact of different interventions on emotions.

## 3 Results

### 3.1 Comparison and analysis results of machine learning models

The performance of K-Nearest Neighbors (KNN), Support Vector Machine (SVM), and Backpropagation Neural Network (BPNN) in recognizing driving stress and anger in terms of accuracy, precision, recall, and F1 score is presented in Table 5.

**TABLE 5:** Comparison of emotion recognition model performance.

Emotion	Model	Accuracy	Precision	Recall	F1 score
Stress	KNN	0.7153	0.5444	0.5178	0.5308
	SVM	0.8056	0.6889	0.7708	0.7275
	BPNN	0.8819	0.8111	0.8318	0.8214
Anger	KNN	0.75	0.6	0.5333	0.5621
	SVM	0.75	0.6117	0.7733	0.6831
	BPNN	0.8333	0.75	0.6939	0.7209

According to the results presented in Table 3, the Backpropagation Neural Network (BPNN) demonstrates exceptional performance in recognizing driving stress and anger. The BPNN model outperforms all other models across all metrics, exhibiting the highest accuracy, precision, recall, and F1 score. Therefore, the BPNN model is selected as the final model for recognizing stress and anger emotions.

The confusion matrix analysis results for the BPNN stress emotion recognition model developed in this study indicate that the model achieved 100% prediction accuracy in both the high Stress and moderate stress categories, with no misclassifications. In the slight stress category, the model correctly identified 67% of the samples, while 33% were misclassified as almost not stress. For the almost not stress category, the model had a correctness rate of 61%, with 39% of samples incorrectly classified as calm. Additionally, the prediction accuracy for the calm stage category also reached 100%. Overall, the model performed well in the emotion recognition task, demonstrating its effectiveness in handling different emotional states.

And the confusion matrix for the BPNN anger emotion recognition mode. In the high anger category, the model achieved a prediction accuracy of 97%, with only 3% misclassified as moderate anger. For the moderate anxious category, 78% of samples were correctly classified, while 22% were misclassified as Slight Anger. In the Slight anger category, the model correctly identified all samples, achieving 100% accuracy. The Almost not anger category also saw a perfect classification rate of 100%. For the Calm category, 22% of the samples were classified as Almost not anger, while 78% were correctly identified as Calm. Overall, the model demonstrates strong performance in identifying emotions, particularly in the Highly Anxious and Slightly Anxious categories, highlighting its effectiveness in emotional recognition tasks.

In the stress emotion recognition model, the input layer comprises six neurons, each corresponding to one of the six features: mean, variance, skewness, kurtosis, root mean square, and power spectral density. The output layer includes five neurons, representing the five classification labels: calm, nearly no stress, slight stress, moderate stress, and high stress. The hidden layer consists of five neurons and was trained over 24 iterations.

In the anger emotion recognition model, the input layer consists of six neurons, each representing one of the six features: mean, variance, skewness, kurtosis, root mean square, and power spectral density. The output layer contains five neurons, corresponding to the five classification labels: calm, nearly no anger, slight anger, moderate anger, and high anger. The hidden layer comprises five neurons and was trained over 29 iterations.

The stress and anger emotion models, classified using the Back Propagation Neural Network (BPNN) algorithm, have been saved in preparation for further regulatory analyses.

### 3.2 Analysis of regulatory effects

#### 3.2.1 Results of stress regulation

**FIGURE 4:**
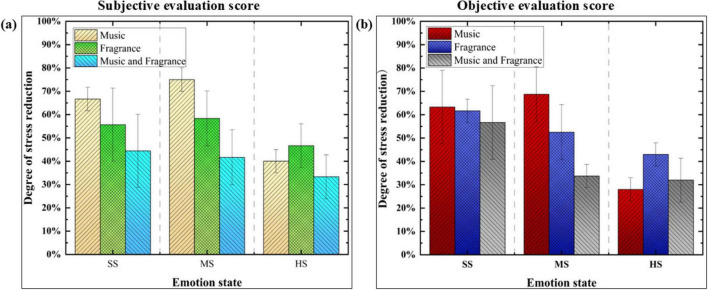
Scores of stress emotion regulation based on subjective and objective data. SS represents “Slight Stress,” MS represents “Moderate Stress,” and HS represents “High Stress.” **(a)** Indicates the subjective evaluation score. **(b)** Indicates the objective evaluation score.

The EEG features obtained from participants exposed to soft music, lemon scent, and the combination of music and scent interventions were input into a pre-trained stress emotion recognition model to obtain classification labels after the intervention. The stress regulation score was calculated by subtracting the post-regulation labels from the pre-regulation labels, which allows us to quantify the effectiveness of the interventions in alleviating stress; specifically, a larger value indicates a greater reduction in stress levels. Additionally, subjective assessment scores collected from the experiment are summarized. Organize as shown in Figure 4.

Figure 4 illustrates the consistency between the subjective ratings of the three regulation methods and the objective EEG data scores in alleviating stress. All three interventions were associated with a reduction in stress levels. calming music was found to be the most effective for alleviating slight stress, while music regulation was the best method for moderating moderate stress. Additionally, lemon fragrance proved to be most effective when combined with other interventions during high stress. The results obtained from the observational data indicate a mutual validation between the subjective and objective measures.

#### 3.2.2 Results of anger regulation

The electroencephalogram (EEG) features collected from participants exposed to calming music, lemon fragrance, and a combination of music and fragrance interventions were input into the pre-trained anger emotion recognition model to derive objective EEG data regulation scores. Additionally, the subjectively collected evaluation scores from the experiment are summarized in Figure 3.

Figure 3 illustrates the consistency between the subjective ratings of the three regulation methods and the objective EEG data scores in alleviating anger. All three interventions were associated with a reduction in anger levels. In conditions of slight anger, lemon fragrance was found to be the most effective. For moderate anger, lemon fragrance alone yielded the best results, while calming music proved to be the most effective intervention for high anger. The results obtained from the observational data indicate a mutual validation between the subjective and objective measures.

### 3.3 Hjorth parameter analysis and validation

To further validate the effects of music and aroma interventions, we conducted a time-frequency analysis of EEG signals across various emotional and stress states. Specifically, we amalgamated slight, moderate, and high-stress conditions into a single stress category, while consolidating slight, moderate, and high-anger conditions into a unified anger category to facilitate analysis.

Our findings indicate a significant increase in the activity parameter under stress conditions, reaching a value of 9.3961, compared to 6.2258 in calm states. This suggests a strong relationship between stress and heightened brain activity. The mobility parameter exhibited minimal variation between the two states, with values of 1.9424 in calm states and 1.8722 during stress. Additionally, the complexity parameter demonstrated a slight increase from 1.5921 in calm states to 1.6432 under stress, reflecting a negligible change overall. In the anger state, the activity level substantially increased to 13.3234, indicating a higher level of physiological activation. Conversely, the mobility and complexity parameters were recorded at 2.0213 and 1.5367, respectively, showing no significant changes when compared to the calm condition.

**FIGURE 5:**
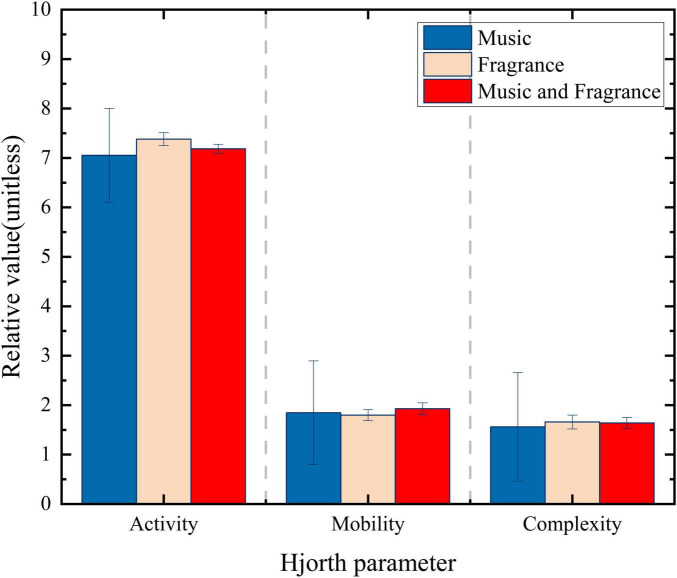
Hjorth parameter of stress emotions.

**FIGURE 6:**
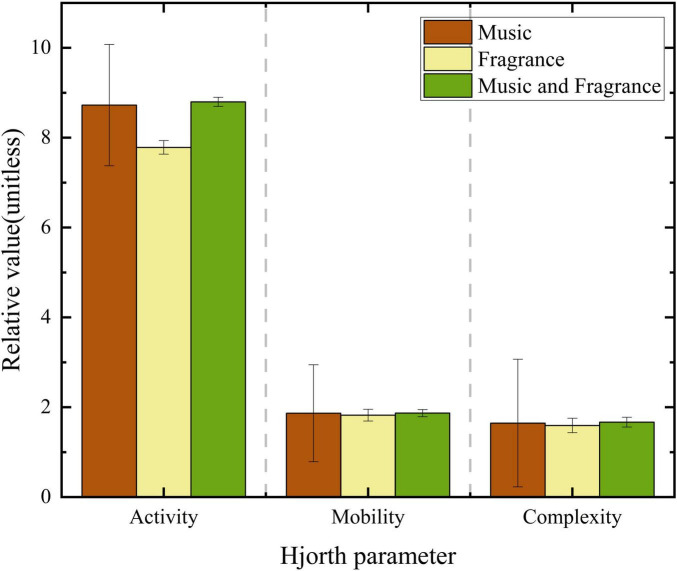
Hjorth parameter of anger emotions.

Next, the activity, mobility and complexity after receiving music, fragrance, and combined regulation were statistically analyzed, as shown in Figures 5, 6.

Observations indicated that the Hjorth activity parameter of the participant, after receiving music, aroma, and combined interventions, was lower compared to the levels observed during tense and emotional states, approaching the activity levels characteristic of calm states.

The analysis of Hjorth parameters indicates that, overall, the use of music alone is the strategy that shows the greatest impact on alleviating stress, while the use of fragrance alone is particularly effective for mitigating anger. These findings further support the role of both music and fragrance in alleviating abnormal emotions and confirm the reliability of the EEG-based models for identifying emotional states, specifically stress and anger, as well as the emotion regulation assessment system. The multidimensional assessment of EEG data enhances the authenticity and accuracy of the results.

## 4 Discussion

This study evaluated the effectiveness of an EEG-based model for identifying abnormal emotions, specifically stress and anger, and introduced music and fragrance as strategies for emotion regulation. Both subjective and objective measures corroborated the effectiveness of these interventions in alleviating drivers’ abnormal emotions. The experimental results demonstrated that the BPNN model exhibited a significant advantage in emotional classification accuracy compared to other commonly used machine learning models. Music was found to be the most effective intervention for slight and moderate stress, while fragrance proved to be more effective during periods of high stress. For slight to moderate anger, fragrance regulation was superior, whereas music was most effective in alleviating high levels of anger.

### 4.1 Discussion on the intervention effect of music and fragrance on emotions

Whether through music, fragrance, or a combination of both, all interventions demonstrate a certain degree of effectiveness in alleviating various emotional states. These findings align with research conducted by Pei et al. (2024) and Cook et al. (2019), which similarly validated the efficacy of music and fragrance in mitigating abnormal emotions.

However, studies by Ning et al. (2022) and Sanyal et al. (2013) indicate that music does not always alleviate abnormal emotions. Certain emotionally charged music, such as sad or intense pieces, may evoke negative feelings or memories, leading to emotional fluctuations and increased anxiety, which can exacerbate stress or anger. Additionally, fast-paced or overly complex music can induce excessive cortical excitation, resulting in physiological responses such as increased heart rate and rapid breathing, potentially hindering emotional relief. In contrast, the gentle music employed in this study effectively reduces heart rate and respiratory frequency, facilitating relaxation and helping to mitigate negative emotions. Similarly, research conducted by Barnes et al. (2018), Wang et al. (2024), and Soars (2009) reveals that not all scents are effective in alleviating drivers’ abnormal emotions; for instance, odors from car engines, body odor, and the scent of new cars can intensify negative feelings. However, the lemon fragrance used in this study enhances driving pleasure, effectively promotes positive emotions, and fosters a sense of inner calm, providing a gentle soothing effect on the nervous system.

### 4.2 The relationship between Hjorth parameters and emotions

The Hjorth parameters are essential tools for signal processing and are widely used in the analysis of electroencephalogram (EEG) signals, particularly in studying emotional states (Fatih et al., 2023). By analyzing Hjorth parameters, researchers can objectively assess and understand the characteristics of EEG activity in drivers experiencing different emotional conditions. This study further employs Hjorth parameter analysis to investigate the effects of music and fragrance on emotional regulation. The results indicate that music alone is the most effective intervention for alleviating stress, while a singular fragrance intervention is most effective in calming anger. Moreover, this study validates the efficacy of both music and fragrance in mitigating abnormal emotions and confirms the reliability of EEG-based models for identifying emotional states (stress and anger) as well as the emotion regulation assessment system.

Hjorth activity serves as an indicator of the intensity of EEG signals and effectively reflects the degree of emotional arousal. In this study, Hjorth activity parameters significantly increased when drivers experienced tension and anger, indicating heightened brain activation in response to abnormal emotions, which aligns with the findings of Mehmood et al. (2022). In contrast, changes in Hjorth mobility and complexity were relatively subtle. This suggests that, while subjects experienced emotional state changes, the effects on EEG signals may be concentrated in specific frequency bands or features, leading to a lack of variability in these parameters.

### 4.3 Limitations and future jobs

This study has several limitations. First, the relatively small sample size may restrict the generalizability and reliability of the findings. Second, the investigation focused exclusively on gentle music and lemon fragrance as stimuli, without examining a broader range of musical genres, scents, and their interactions with various emotional states. Importantly, we did not perform statistical hypothesis testing to confirm the stimulus effects due to the exploratory nature and sample size constraints of this study; the observed effects should therefore be interpreted as preliminary trends requiring future validation. Additionally, while EEG provides direct measurements of brain activity, the complexity and multidimensional nature of emotions require the incorporation of additional physiological indicators (such as heart rate variability) and behavioral data to comprehensively assess the impact of music and analyze the predictive capacity of various signal combinations. Future research will aim to develop more refined modeling frameworks and optimization methods to enhance predictive accuracy, including rigorous statistical verification of stimulus effects through controlled experimental designs with adequate power.

## 5 Conclusion

This study developed an EEG-based model for recognizing abnormal emotions, revealing the differential regulatory effects of music, fragrance, and their combined strategies on drivers’ tension and anger. The results indicate significant heterogeneity in responses to regulatory strategies based on various types and levels of emotions, with a consistent relationship between subjective experiences and EEG characteristics. This not only provides a scientific basis for managing driver emotions but also opens potential avenues for developing personalized emotion regulation systems in smart cabins. Future research could further integrate multimodal physiological data (such as heart rate and skin conductance) and optimize real-time emotion monitoring algorithms for dynamic driving scenarios. Additionally, exploring the adaptability of regulatory strategies across different cultural backgrounds and individual characteristics could enhance both driving safety and traffic efficiency.

## Data Availability

The original contributions presented in this study are included in this article/supplementary material, further inquiries can be directed to the corresponding author.

## References

[B1] Âbele L. Haustein S. Møller M. Zettler I. (2020). Links between observed and self-reported driving anger, observed and self-reported aggressive driving, and personality traits. *Accid. Anal. Prev.* 140:105516. 10.1016/j.aap.2020.105516 32244089

[B2] Alazrai R. Abuhijleh M. Alwanni H. Daoud M. I. (2019). A deep learning framework for decoding motor imagery tasks of the same hand using EEG signals. *IEEE Access* 7 109612–109627. 10.1109/ACCESS.2019.2934018

[B3] Atkinson J. Campos D. (2016). Improving BCI-based emotion recognition by combining EEG feature selection and kernel classifiers. *Expert Syst. Appl.* 47 35–41. 10.1016/j.eswa.2015.10.049

[B4] Bai B. Zhang J. Wu X. Wei Zhu G. Li X. (2021). Reliability prediction-based improved dynamic weight particle swarm optimization and back propagation neural network in engineering systems. *Expert Syst. Appl.* 177:114952. 10.1016/j.eswa.2021.114952

[B5] Barnes N. M. Ng T. W. Ma K. K. Lai K. M. (2018). In-cabin air quality during driving and engine idling in air-conditioned private vehicles in Hong Kong. *Int. J. Environ. Res. Public Health* 15:611. 10.3390/ijerph15040611 29584686 PMC5923653

[B6] Bobermin M. P. Silva M. M. Ferreira S. (2021). Driving simulators to evaluate road geometric design effects on driver behaviour: A systematic review. *Accid. Anal. Prevent*. 150:105923. 10.1016/j.aap.2020.105923 33307477

[B7] Celiñski I. (2022). “Problems of studies on emotions in road traffic,” in *Proceedings of the scientific and technical conference transport systems theory and practice*, (Cham: Springer), 120–140.

[B8] Chaichanasittikarn O. Jiang M. Seet M. Saba M. Hamano J. Dragomir A. (2023). “Wearable EEG-based classification of odor-induced emotion,” in *Proceedings of the 2023 11th international IEEE/EMBS conference on neural engineering (NER)*, (Piscataway, NJ: IEEE), 1–4.

[B9] Conceição M. A. Monteiro M. M. Kasraian D. Van Den Berg P. Haustein S. Alves I. (2023). The effect of transport infrastructure, congestion and reliability on mental wellbeing: A systematic review of empirical studies. *Trans. Rev.* 43 264–302. 10.1080/01441647.2022.2100943

[B10] Cook T. Roy A. R. Welker K. M. (2019). Music as an emotion regulation strategy: An examination of genres of music and their roles in emotion regulation. *Psychol. Music* 47 144–154. 10.1177/030573561773462

[B11] Das R. K. Martin A. Zurales T. Dowling D. Khan A. (2023). A survey on EEG data analysis software. *Science* 5:23. 10.3390/sci5020023

[B12] Dixon M. L. Thiruchselvam R. Todd R. Christoff K. (2017). Emotion and the prefrontal cortex: An integrative review. *Psychol. Bull.* 143:1033. 10.1037/bul0000096 28616997

[B13] Fatih N. Wibawa A. D. Purnomo M. H. Mas A. (2023). “Comparative analysis of EEG-based emotion recognition between male and female participants using Hjorth parameter,” in *Proceedings of the 2023 international electronics symposium (IES)*, (New York, NY: IEEE), 179–185.

[B14] Godfrey H. D. (2018). *Essential oils for mindfulness and meditation: Relax, replenish, and rejuvenate.* Rochester, VT: Healing Arts Press.

[B15] Habibifar N. Salmanzadeh H. (2022). Relationship between driving styles and biological behavior of drivers in negative emotional state. *Trans. Res. F Traffic Psychol. Behav.* 85 245–258. 10.1016/j.trf.2022.01.010

[B16] Han L. Du Z. Wang S. He S. (2024). The effects of tunnel radius, turn direction, and zone characteristics on drivers’ visual performance. *Tunnell. Underground Space Technol.* 152:105912. 10.1016/j.tust.2024.105912

[B17] Hasan M. Yasmin S. Pias T. S. (2021). “Fine-grained emotion recognition from EEG signal using fast Fourier transformation and CNN,” in *Proceedings of the 2021 joint 10th international conference on informatics, electronics & vision (ICIEV) and 2021 5th international conference on imaging, vision & pattern recognition (icIVPR)*, (Berlin: IEEE), 1–9.

[B18] Hu Z. Lou S. Xing Y. Wang X. Cao D. Lv C. (2022). Review and perspectives on driver digital twin and its enabling technologies for intelligent vehicles. *IEEE Trans. Intell. Vehicles* 7 417–440. 10.1109/TIV.2022.3195635

[B19] Islam M. K. Rastegarnia A. Yang Z. (2016). Methods for artifact detection and removal from scalp EEG: A review. *Neurophysiol. Clin.* 46 287–305. 10.1016/j.neucli.2016.07.002 27751622

[B20] Khalili Z. Moradi M. H. (2009). “Emotion recognition system using brain and peripheral signals: Using correlation dimension to improve the results of EEG,” in *Proceedings of the 2009 international joint conference on neural networks*, (Atlanta, GA: IEEE), 1571–1575.

[B21] Laktionova T. Kvasha I. Voznessenskaya V. (2024). Male body odor affects emotional state, LH, and cortisol secretion in women of different age groups. *Brain Sci.* 14:721. 10.3390/brainsci14070721 39061461 PMC11274401

[B22] Li G. Yuan Y. Ouyang D. Zhang L. Yuan B. Chang X. (2023). Driver distraction from the EEG perspective: A review. *IEEE Sens. J.* 24 2329–2349. 10.1109/JSEN.2023.3339727

[B23] Li J. Ge Y. Yu T. Qu W. (2023). Social exclusion and dangerous driving behavior: The mediating role of driving anger and moderating role of cognitive reappraisal. *Curr. Psychol.* 42 21667–21680. 10.1007/s12144-022-03259-9

[B24] Li X. Song D. Zhang P. Zhang Y. Hou Y. Hu B. (2018). Exploring EEG features in cross-subject emotion recognition. *Front. Neurosci.* 12:162. 10.3389/fnins.2018.00162 29615853 PMC5867345

[B25] Liao M. (2024). Analysis of the causes, psychological mechanisms, and coping strategies of short video addiction in China. *Front. Psychol*. 15:1391204. 10.3389/fpsyg.2024.1391204 39165759 PMC11333346

[B26] Lin Y. Lin H. Lu Z. (2022). “Warning of dangerous driving behavior caused by drivers and road environmental factors,” in *Proceedings of CECNet 2022*, (Amsterdam: IOS Press).

[B27] Mehmood R. M. Bilal M. Vimal S. Lee S.-W. (2022). EEG-based affective state recognition from human brain signals by using Hjorth-activity. *Measurement* 202:111738. 10.1016/j.measurement.2022.111738

[B28] Mukherjee A. Anwaruzzaman A. (2024). Gridlock gloom: A geographical analysis of commuters’ perceptions on traffic congestion. *Int. J. Hum. Capit. Urban Manag.* 9 617–636. 10.22034/IJHCUM.2024.04.05 36190387

[B29] Ning M. Wen S. Zhou P. Zhang C. (2022). Ventral tegmental area dopaminergic action in music therapy for post-traumatic stress disorder: A literature review. *Front. Psychol.* 13:1014202. 10.3389/fpsyg.2022.1014202 36300072 PMC9589351

[B30] Pedroni A. Bahreini A. Langer N. (2019). Automagic: Standardized preprocessing of big EEG data. *Neuroimage* 200 460–473. 10.1016/j.neuroimage.2019.06.046 31233907

[B31] Pei S. Chen J. Lu J. Yao L. Zhang N. (2024). Exploring the physiological response differences of β-caryophyllene, linalool and citral inhalation and their anxiolytic potential. *Heliyon* 10:e38941. 10.1016/j.heliyon.2024.e38941 39430514 PMC11490826

[B32] Peng Y. Xu Q. Lin S. Wang X. Xiang G. Huang S. (2022). The application of electroencephalogram in driving safety: Current status and future prospects. *Front. Psychol.* 13:919695. 10.3389/fpsyg.2022.919695 35936295 PMC9354986

[B33] Pring E. X. Olsen K. N. Mobbs A. E. Thompson W. F. (2024). Music communicates social emotions: Evidence from 750 music excerpts. *Sci. Rep.* 14:27766. 10.1038/s41598-024-78156-1 39532962 PMC11557968

[B34] Putkinen V. Zhou X. Gan X. Yang L. Becker B. Sams M. (2024). Bodily maps of musical sensations across cultures. *Proc. Natl. Acad. Sci. U.S.A.* 121 e2308859121. 10.1073/pnas.2308859121 38271338 PMC10835118

[B35] Richard L. Charbonneau D. (2009). An introduction to E-Prime. *Tutor. Q. Methods Psychol.* 5 68–76. 10.20982/tqmp.05.2.p068

[B36] Sanyal T. Kumar V. Nag T. C. Jain S. Sreenivas V. Wadhwa S. (2013). Prenatal loud music and noise: Differential impact on physiological arousal, hippocampal synaptogenesis and spatial behavior in one day-old chicks. *PLoS One* 8:e67347. 10.1371/journal.pone.0067347 23861759 PMC3702537

[B37] Sari D. A. L. Kusumaningrum T. D. Faqih A. Kusumoputro B. (2019). “Emotion classification system based on non-linear EEG signal using backpropagation neural network,” in *Proceedings of the AIP conference*, (Melville, NY: AIP Publishing).

[B38] Shashidhar R. Kadakol P. Sreeniketh D. Patil P. Krishnappa K. H. Madhura R. (2023). “EEG data analysis for stress detection using k-nearest neighbor,” in *Proceedings of the 2023 international conference on integrated intelligence and communication systems (ICIICS)*, (Bhopal: IEEE), 1–7.

[B39] Sikander G. Anwar S. (2018). Driver fatigue detection systems: A review. *IEEE Trans. Intell. Trans. Syst.* 20 2339–2352. 10.1109/TITS.2018.2868499

[B40] Smith A. Nicholson-Lord K. (2024). Effects of a lemon aroma on attention, reaction time and mood. *World J. Pharm. Res.* 13 840–858. 10.20959/wjpr20244-31747

[B41] Soars B. (2009). Driving sales through shoppers’ sense of sound, sight, smell and touch. *Int. J. Retail Distrib. Manage*. 37, 286–298.

[B42] Son G. Kim Y. (2021). EEG-based emotion classification for verifying the korean emotional movie clips with support vector machine (SVM). *Complexity* 2021:5497081. 10.1155/2021/5497081

[B43] Subasi A. Tuncer T. Dogan S. Tanko D. Sakoglu U. (2021). EEG-based emotion recognition using tunable Q wavelet transform and rotation forest ensemble classifier. *Biomed. Signal Process. Control* 68:102648. 10.1016/j.bspc.2021.102648

[B44] Verdonschot M. Van Steenbergen H. Spapé R. (2019). *The E-primer: An introduction to creating psychological experiments in E-Prime.* Leiden: Leiden University Press.

[B45] Wagh K. P. Vasanth K. (2022). Performance evaluation of multi-channel electroencephalogram signal (EEG) based time frequency analysis for human emotion recognition. *Biomed. Signal Process. Control* 78:103966. 10.1016/j.bspc.2022.103966

[B46] Wang C. Lin Y. Ptukhin Y. Liu S. (2024). Air quality in the car: How CO_2_ and body odor affect drivers’ cognition and driving performance? *Sci. Total Environ.* 911:168785. 10.1016/j.scitotenv.2023.168785 37996033

[B47] Wu Y. Li W. Zhang J. Tang B. Xiang J. Li S. (2023). Driver’s hand-foot coordination and global-regional brain functional connectivity under fatigue: Via graph theory and explainable artificial intelligence. *IEEE Trans. Intell. Veh*. 9, 3493–3508.

[B48] Xi J. Wang P. Ding T. Tian J. Li Z. (2022). Mental health and safety assessment methods of bus drivers. *Appl. Sci.* 13:100. 10.3390/app13010100

[B49] Xiao Y. Sun J. Tao G. (2024). Effects of soothing music on the intraoperative management of patients undergoing tension-free herniorrhaphy: A retrospective study. *Noise Health* 26 198–204. 10.4103/nah.nah_5_24 38904823 PMC11530102

[B50] Xu H. Wu Y. Hamari J. (2024). Musical atmosphere as a (dis)tractive facet of user interfaces: An experiment on sustainable consumption decisions in eCommerce. *Int. J. Inform. Manage*. 75:102715. 10.1016/j.ijinfomgt.2023.102715

[B51] Zhang F. Xue H. Zhang Y. (2019). A new BP neural network fusion algorithm for multi-source remote sensing data on groundwater. *Appl. Ecol. Environ. Res.* 17:23. 10.15666/aeer/1704_90839095

