# Correction: Research on auditory and olfactory regulation methods for abnormal driver emotions based on EEG signals

**DOI:** 10.3389/fnhum.2025.1647983

**Published:** 2025-07-01

**Authors:** Bangbei Tang, Yan Li, Yingzhang Wu, Yilun Li, Qizong Yue

**Affiliations:** ^1^School of Intelligent Manufacturing Engineering, Chongqing University of Arts and Sciences, Chongqing, China; ^2^Department of Physiology, Army Medical University, Chongqing, China; ^3^School of Vehicle and Mobility, Tsinghua University, Beijing, China; ^4^School of Music and Dance, Henan Institute of Science and Technology, Xinxiang, China; ^5^China Music Mental Health Institute, Southwest University, Chongqing, China

**Keywords:** brain, driving emotions, music intervention, emotional regulation, fragrance intervention

Author Qizong Yue was erroneously spelled as Qǐóng Yué.

Affiliation 3 School of Vehicle and Mobility, Tsinghua University, Beijing, China was erroneously given as 3 School of Vehicle and Mobility, Tsinghua University, Chongqing, China.

The original version of this article has been updated.

